# Robust HIV-specific CD4^+^ and CD8^+^ T-cell responses distinguish elite control in adolescents living with HIV from viremic nonprogressors

**DOI:** 10.1097/QAD.0000000000003078

**Published:** 2021-10-01

**Authors:** Vinicius A. Vieira, Jane Millar, Emily Adland, Maximilian Muenchhoff, Julia Roider, Claudia Fortuny Guash, Denise Peluso, Beatriz Thomé, Maria C. Garcia-Guerrero, Mari C. Puertas, Alasdair Bamford, Christian Brander, Mary Carrington, Javier Martinez-Picado, John Frater, Gareth Tudor-Williams, Philip Goulder

**Affiliations:** a Department of Paediatrics, University of Oxford, Oxford, UK; b HIV Pathogenesis Programme, Doris Duke Medical Research Institute, University of KwaZulu-Natal (UKZN), Durban, South Africa; c Max von Pettenkofer-Institute, Department of Virology, Ludwig-Maximilians-University; d German Center for Infection Research (DZIF); e Department of Infectious Diseases, Ludwig-Maximilians-University, Munich, Germany; f *Unidad* de Enfermedades Infecciosas, Servicio de Pediatría, Hospital Sant Joan de Déu, Universitat de Barcelona, Barcelona, Spain; g Instituto de Infectologia Emílio Ribas; h Universidade Federal de São Paulo, Escola Paulista de Medicina, Departamento de Medicina Preventiva, São Paulo, Brazil; i IrsiCaixa - AIDS Research Institute, Badalona, Spain; j Great Ormond Street Hospital for Children NHS Foundation Trust; k UCL Great Ormond Street Institute of Child Health, London, UK; l Universitat de Vic-Universitat Central de Catalunya, Vic; m Institució Catalana de Recerca i Estudis Avançats, Barcelona, Spain; n Ragon Institute of Massachusetts General Hospital, Massachusetts Institute of Technology, and Harvard, Cambridge, Massachusetts; o Basic Science Program, Frederick National Laboratory for Cancer Research in the Laboratory of Integrative Cancer Immunology, Bethesda, Maryland, USA; p Nuffield Department of Medicine, University of Oxford; q Oxford NIHR Biomedical Research Centre, Oxford; r Department of Infectious Diseases, Imperial College, London, UK.

**Keywords:** elite controllers, immune activation, pediatrics, T-cell response, viral control

## Abstract

**Background:**

Elite controllers are therapy-naive individuals living with HIV capable of spontaneous control of plasma viraemia for at least a year. Although viremic nonprogressors are more common in vertical HIV-infection than in adults’ infection, elite control has been rarely characterized in the pediatric population.

**Design:**

We analyzed the T-cell immunophenotype and the HIV-specific response by flow cytometry in four pediatric elite controllers (PECs) compared with age-matched nonprogressors (PNPs), progressors and HIV-exposed uninfected (HEUs) adolescents.

**Results:**

PECs T-cell populations had lower immune activation and exhaustion levels when compared with progressors, reflected by a more sustained and preserved effector function. The HIV-specific T-cell responses among PECs were characterized by high-frequency Gag-specific CD4^+^ T-cell activity, and markedly more polyfunctional Gag-specific CD8^+^ activity, compared with PNPs and progressors. These findings were consistently observed even in the absence of protective HLA-I molecules such as HLA-B∗27/57/81.

**Conclusion:**

Pediatric elite control is normally achieved after years of infection, and low immune activation in PNPs precedes the increasing ability of CD8^+^ T-cell responses to achieve immune control of viraemia over the course of childhood, whereas in adults, high immune activation in acute infection predicts subsequent CD8^+^ T-cell mediated immune control of viremia, and in adult elite controllers, low immune activation is therefore the consequence of the rapid CD8^+^ T-cell mediated immune control generated after acute infection. This distinct strategy adopted by PECs may help identify pathways that facilitate remission in posttreatment controllers, in whom protective HLA-I molecules are not the main factor.

## Introduction

Vertically HIV-infected children usually progress faster to AIDS than adults in the absence of antiretroviral therapy (ART). However, viremic long-term nonprogressors, who maintain normal-for-age CD4^+^ T-cell counts despite viremia, are more common in the pediatric population [1]. Around 10% of ART-naive HIV-infected infants maintain a normal CD4^+^ T-cell count for years via mechanisms that mimic those observed in nonpathogenic simian immunodeficiency virus (SIV) infection in nonhuman primates, with low immune activation and low CCR5 expression in long-lived CD4^+^ T-cell subsets, despite ongoing viral replication [2,3]. By contrast, spontaneous control of viraemia is extremely rare in children [4].

Defined as a minimum of three consecutive undetectable plasma HIV-RNA loads (< 50 copies/ml) spanning at least a year in the absence of ART [5], pediatric elite controllers (PECs) have therefore been scarcely reported in the literature. We recently described a group of 11 PECs from different cohorts, with an estimated prevalence of 0.08%, at least five times lower than in adults [4]. As in adult elite control [6], PECs are strongly biased towards the female sex (10 of the 11) and, in contrast to adult elite controllers, who usually achieve viremic control within the first year after seroconversion [7], elite control was only achieved after a median of 6.5 years of infection [4]. Here, we sought to investigate the activation and exhaustion marker expression on T-cell memory subsets, and the HIV-specific T-cell response in this rare phenotype of elite control in vertically infected adolescents compared with viremic noncontrollers, progressors, and HIV-exposed uninfected (HEUs) adolescents.

## Materials and methods

### Study population

PEC was defined as vertically HIV-infected ART-naive children/adolescents with a minimum of three consecutive plasma HIV-RNA loads of less than 50 copies/ml spanning at least 1 year [5,6]. Cryopreserved peripheral blood mononuclear cells (PBMCs) were obtained from four PECs during the period of viremic control. For comparison, we matched by age a group of pediatric nonprogressors (PNPs, *n* = 13), pediatric progressors (*n* = 10), and HEUs (*n* = 9). No data were available on plasma viral load and drug suppression of HEU's mothers. PNPs were defined as ART-naive individuals with an absolute CD4^+^ T-cell count above 750 cells/μl and pediatric progressors as an ART-naive with an absolute count below 350 cell/μl. All HIV-infected participants were diagnosed before the age of 10 years, and also, in every case wherein the maternal HIV status could be ascertained, were born to a mother with HIV-infection, consistent with vertical transmission.

All participants living with HIV were born in sub-Saharan Africa countries and receive clinical care in out-patient clinics and are now on ART or opted to remain ART-naive, despite medical advice. PEC-1 receives care at St Mary's Hospital (London UK, country of origin: Ghana), and PEC-2 and PEC-3 are followed at Sant Joan de Déu Children's Hospital (Barcelona, Spain, country of origin: Nigeria and Ethiopia, respectively). PEC-4 and all PNPs, pediatric progressors, and HEUs are followed at Prince Mshiyeni Memorial Hospital (Durban, South Africa). All participants or their caregivers for underage children provided informed consent at enrolment. The study was approved by the Institutional Review Boards or equivalents in each contributing site.

### Peripheral blood mononuclear cells processing and immunophenotype

Isolation of PBMCs was made by Ficoll density gradient centrifugation and cells were stored in liquid nitrogen. PBMCs were thawed, counted, and 1 million cells were rested in R10 medium for 3 h at 37°C in 5% CO_2_ before surface staining. Then, cells were washed and stained with Live/Dead near-IR stain (Invitrogen Corporation, Frederick, Maryland, USA) according to the manufacturer instructions. After 30 min at room temperature, cells were washed again and incubated for another 30 min at 4°C in FACS Buffer containing the antibodies against CD3 (BV605, UCHT1; Biolegend, San Diego, California, USA), CD4 (BV650, RPA-T4, Biolegend), CD8a (BV570, RPA-T8; Biolegend), CD45RA (AlexaFluor700, H100; Biolegend), CCR7 (Pacific Blue, G043H7; Biolegend), CD27 (BV510, M-T271; Biolegend), CCR5 (PE-Cy7, HM-CCR5; Biologend), HLA-DR (APC-R700, G46-6, BD), CD38 (PerCP/Cy5.5, HIT2; Biolegend), CD39 (APC, A1; eBioscience), CD73 (PE, AD-2; Biolegend), and PD-1 (PE-eFluor610, J105; eBioscience). Cells were washed and fixed in 2% paraformaldehyde (PFA) and acquired on a BD LSR II.

### Intracellular cytokine stimulation assay

Thawed PBMCs were rested in R10 overnight at 37°C in 5% CO_2_. Cells were stimulated with Gag, Env, and Nef pools of overlapping HIV-1 clade C consensus peptides (NIH AIDS Reagent Program) at a final concentration of 2 μg/ml for each peptide in the presence of anti-CD28/anti-CD49d at 1 μg/ml (BD) and anti-CD107a (PE-Cy7, H4A3; Biolegend). PMA/Ionomycin cocktail (Biolegend) and 0.2% DMSO were used as a positive and a negative control, respectively. Cells were incubated for 6 h and Brefeldin A at 5 μg/ml (Biolegend) and Monensin at 1 μg/ml (Biolegend) were added after 1 h of stimulation outset. Stimulated cells were washed and stained with Live/Dead near-IR stain (Invitrogen) for 30 min at room temperature. Then, cells were washed again and incubated for another 30 min at 4°C with antibodies against CD3, CD4, and CD8 as above. Cells were fixed and permeabilized with Cytofix/Cytoperm solution (BD) for 45 min at 4°C and stained with antibodies against IFN-γ (PE-Dazzle594, B27; Biolegend), IL-2 (BV510, MQ1–17H12; Biolegend), TNF-α (AlexaFluor700, MAb11; Biolegend), MIP-1β (PE, D21–1351; BD, San Diego, California, USA), Perforin (FITC, B-D48; Biolegend), and Granzyme B (APC, GB11; Invitrogen) for 30 min at 4°C. Cells were washed and acquired on a BD LSR II.

### FACS analyses

The data were analyzed in FlowJo v10.6.2 (Tree Star LLC, Ashland, Oregon, USA). The gating strategy is detailed in Supplemental Figures 1 and 2. Positive gates were selected using Fluorescence Minus One (FMO) and memory subsets were obtained using Boolean Gates. For Intracellular cytokine stimulaiton (ICS) assay, positive responses were considered if at least three times above the negative control.

### Quantification of total HIV-DNA

Total HIV-DNA was measured from using droplet digital PCR (ddPCR; BioRad, Hercules, California, USA) with 5′LTR or gag primers and probes, as previously described [8]. Briefly, after thawing, at least 2 million total PBMC were incubated overnight in lysis buffer. DNA copy numbers were calculated using the QuantaSoft (BioRad) software and normalized to the number of input cells per reaction using quantification of the RPP30 single copy gene to express the result as copies per million PBMC. The limit of detection for each sample was estimated according to the total input cell number. Total HIV-DNA copies were further adjusted to 1.0 million CD4^+^ T-cell according to the CD4^+^ T-cell frequency in each sample, as analyzed by flow cytometry.

### Human leukocyte antigen class I type

High-resolution genotyping for HLA-A, HLA-B, and HLA-C was determined by PCR sequence-based typing. HLA sequences were analyzed using the ASSIGN software (Conexio Genomics, Fremantle, Western Australia, Australia). In addition to the four PECs described, we were able HLA type three other PECs followed at Hospital Emílio Ribas (Sao Paulo, Brazil) and Great Ormond Street Hospital (London, UK) (Supplemental Figure 3). They are part of the PEC cohort we previously described [4]. However, due to the absence of samples during the period of elite control, these three individuals were not included in the T-cell compartment assessment undertaken here.

### Statistical analyses

Statistical comparison between three and four groups was done using Kruskal--Wallis’ test expressed by the exact *P* value due to the small sample size using GraphPad Prism version 9.0 (GraphPad Software Inc., San Diego, California, USA). When applied, it was followed by Dunn's test to correct for multiple comparisons. Spearman was used to show correlation and a simple linear regression model for best-fit line and 95% confidence interval. The data are expressed in median and interquartile ranges. The polyfunctional profiles shown in the pie charts were done with the permutation test performed by Spice version 6.0. The expression levels on heatmap are shown in median *z*-scores and the figure built using the *pheatmap* package in R (R Foundation, Vienna, Austria). Principal component analysis (PCA) was performed using the *ggbiplot* package. The correlation matrix was built with the *corrplot* package.

## Results

**Fig. 1:**
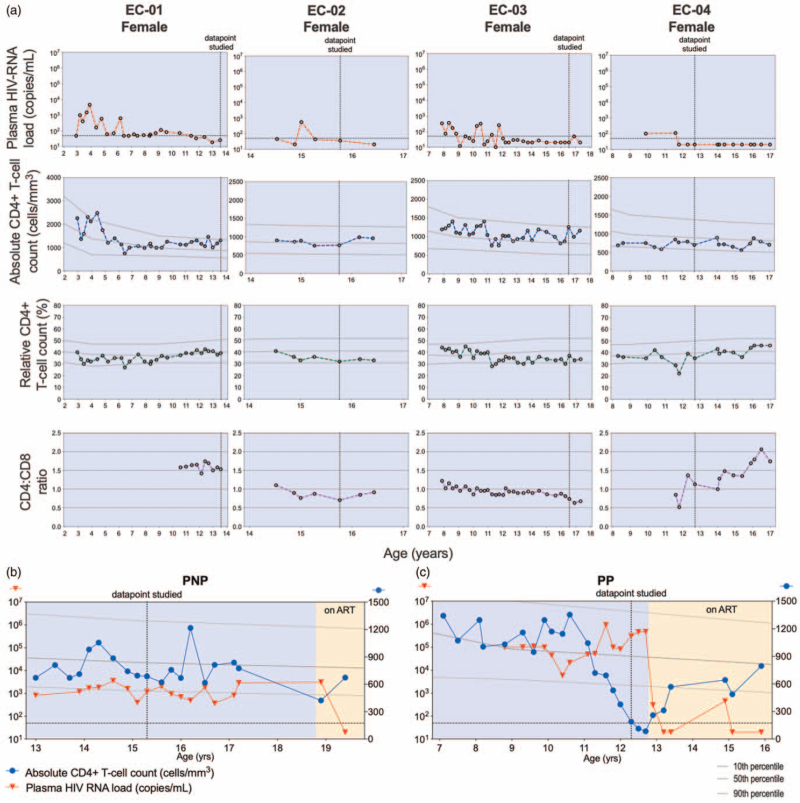
Longitudinal clinical data in different diseases phenotypes of pediatric HIV-infection.

To evaluate the T-cell compartment in PECs, we investigated four PECs (median age 14.6 years, range 12.7–16.6 years) during their period of viremic control (Fig. 1a) and compared with age-matched PNPs [median 13.6 years, interquartile range (IQR) 12.2–15.7, Fig. 1b], pediatric progressors (median 13.0 years, IQR 11.3–15.0, Fig. 1c), and HEUs (median 14.5 years, IQR 12.0–16.6). The clinical characteristics are summarized in Supplemental Table 1. As expected, PECs tended to have a lower total HIV-DNA load (median 286 total HIV-DNA copies/10^6^ CD4^+^ T-cells) when compared with the PNPs (median 7244, *P* = 0.12) and to the pediatric progressors (median 22 574, *P* = 0.0029) (Supplemental Figure 4A). When combining the three groups, total HIV-DNA copies correlated directly with plasma HIV-RNA copies, and indirectly with absolute and relative (not shown) CD4^+^ T-cell count and CD4:CD8 ratio (Supplemental Figure 4B-D).

(a) Plasma HIV-RNA load, absolute and relative CD4^+^ T-cell count, and CD4:CD8 ratio are shown for the four pediatric elite controllers (PECs). (b,c) Representative examples for longitudinal plasma HIV-RNA (red triangles) and absolute CD4^+^ T-cell count (blue circles) of a pediatric non-progressor (PNP) (b) and a pediatric progressor (PP) (c). The vertical dashed line represents the datapoint selected throughout this study. The horizontal dashed line represents the plasma HIV-RNA of 50 copies/ml. The 10th, 50th, and 90th percentiles for absolute and percentage CD4^+^ T-cell for HIV-uninfected children are represented by the three grey lines.

**Fig. 2:**
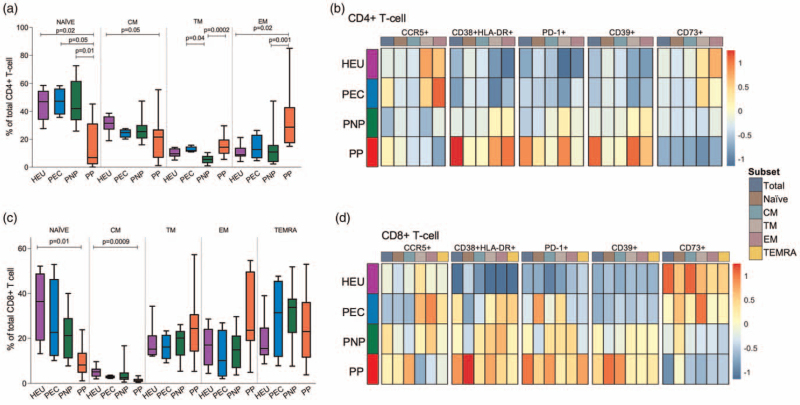
Low levels of T-cell activation and differentiation in PECs.

The CD4^+^ T-cell populations in PECs were distributed towards a higher proportion of naive cells, similar to those observed in the HEUs and the PNPs, while the effector memory population was expanded in the pediatric progressors (Fig. 2a). Consistent with this pattern, the level of immune activation (HLA-DR+CD38+) on central memory and transitional memory was significantly lower in PECs and comparable to HEUs (Fig. 2b and Supplemental Table 2). PD-1 and CD39 were also more highly expressed on CD4^+^ T-cell of pediatric progressors when compared with HEUs. As expected, CCR5 on total CD4^+^ T-cells did not differ markedly between groups but was expressed at higher levels on the short-lived effector memory subsets in the PECs and the HEUs (Fig. 2b).

(a) Frequency of CD4^+^ T-cell subsets defined as naive (CD45RA+CCR7+CD27+, central memory (CM, CD45RA-CCR7+CD27+), transitional memory (TM, CD45RA-CCR7-CD27+), and effector memory (EM, CD45RA-CCR7-CD27-) in HIV-exposed uninfected (HEU) (purple), pediatric elite controller (PEC) (blue), pediatric non-progressor (PNP) (green), and pediatric progressor (PP) (red). The data are shown in median and interquartile. Comparisons were done with Kruskal--Wallis test followed by Dunn's test to correct for multiple comparisons. (^∗^*P* < 0.05; ^∗^*P* < 0.01; ^∗∗∗^*P* < 0.001). B. Heatmap showing the median z-score of the frequency of CCR5+, HLA-DR+CD38+, PD-1+, CD39+, and CD73+ for total and each CD4^+^ T-cell subset in each group. (c) Percentage of CD8^+^ T-cell subsets defined as naive, CM, TM, EM, and terminal effector memory (TEMRA, CD45RA+CCR7-CD27-) in HEU, PEC, PNP, and PP. (d) Same as B but for CD8^+^ T cells.

By contrast to the less terminally differentiated phenotype of CD4^+^ T cells in the PEC, the CD8^+^ T-cell compartment in all groups with individuals living with HIV exhibited a pattern typical of chronic viral infection. There was a progressive decrease of the naive population and a corresponding expansion of the effector subsets of transitional memory, effector memory, and terminal effector memory (TEMRA) in these groups, markedly so in the pediatric progressors (Fig. 2c). Interestingly, both PNPs and pediatric progressors had a higher frequency of activated and exhausted (PD-1+) cells compared with HEUs, but not PECs (Fig. 2d and Supplemental Table 3). Again, all individuals living with HIV, but not PECs, expressed higher levels of CD39 and lower levels of CD73 compared with HEUs.

**Fig. 3:**
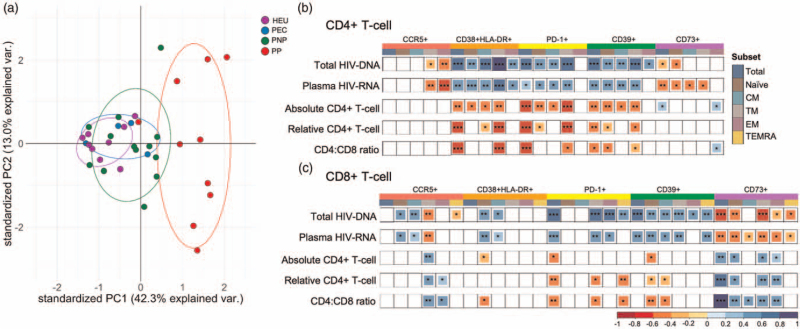
Multivariate analysis of T-cell exhaustion and memory differentiation in different disease phenotypes.

To further visualize immune differences between the four groups, a PCA was undertaken using markers of differentiation, activation, and exhaustion (Fig. 3a). Undeniable confounders such as total HIV-DNA viral load, plasma HIV-RNA viral load, and CD4^+^ T-cell count were not included in this initial PCA analysis. The pediatric progressors clustered in a distinct and a broader group, while the PECs grouped closer to the HEUs and PNPs. The main variables and their loading coefficients that explain the variations observed in PC1 were HLA-DR+CD38+ (0.8523), PD-1+ (0.8014), and CD39+ (0.7683) in CD4^+^ T cells, and CD73+ (-0.8392), PD-1+ (0.7603), and naive cell markers (-0.8245) in CD8^+^ T cells. Indeed, the expression of activation and exhaustion markers on CD4^+^ and CD8^+^ T cells positively correlated with total HIV-DNA and plasma HIV-RNA copies, and inversely correlated with absolute and relative CD4^+^ T-cell count, and CD4:CD8 ratio. Lower total HIV-DNA was associated with CD73 expression on both CD4^+^ and CD8^+^ T-cell populations (Fig. 3b and c, Supplemental Figure 5).

(a) Principal component analysis (PCA) was conducted with markers of activation and exhaustion on CD4^+^ and CD8^+^ T cells to visualize the difference between the groups. The two PCA axes selected explain most of the variations found. (b,c) Correlation of total HIV-DNA and plasma HIV-RNA copies, absolute and relative CD4^+^ T-cell counts, and CD4:CD8 ratio with the different markers and subsets of CD4^+^ (b) and CD8^+^ (c) T-cell are shown for those statistically significant. Layers of red indicate a negative correlation and layers of blue indicate a positive correlation. (^∗^*P* < 0.05; ^∗∗^*P* < 0.01; ^∗∗∗^*P* < 0.001).

**Fig. 4:**
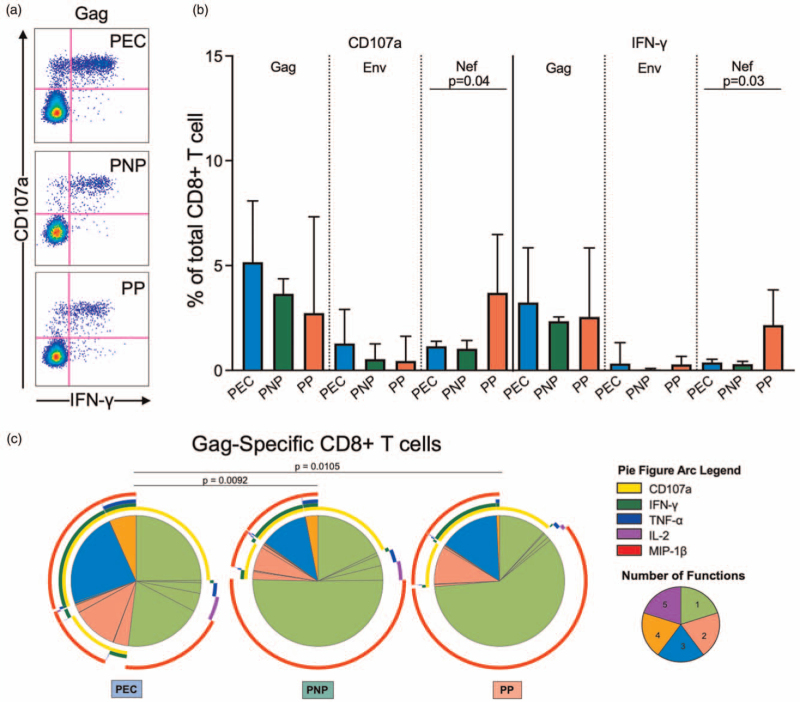
HIV-specific response of CD8^+^ T cells.

Next, we investigated the role of the HIV-specific T-cell response in control of viremia among PECs. Interestingly, only two of the seven PECs who have been HLA Class I typed expressed one of HLA-B∗27/57/58 : 01/81 : 01 described as protective in adult elite controllers (Supplemental Table 4) [9–12]. Robust IFN-γ and degranulation (CD107a+) CD8^+^ T-cell responses were observed in PECs upon stimulation with Gag, but neither these markers nor IL-2, TNF-α, MIP-1β, Granzyme B, and Perforin expression (Supplemental Figure 6A and B) differed in magnitude from the Gag-specific responses observed in PNPs and pediatric progressors (Fig. 4a and b). However, the Gag-specific CD8^+^ T-cells were markedly more polyfunctional in PECs, expressing more two, three, or four functions simultaneously (*P* = 0.0092 and *P* = 0.0105, when compared with PNPs and pediatric progressors, respectively) (Fig. 4c). Of note, pediatric progressors also had significantly higher magnitude Nef-specific CD8^+^ T-cell responses (Fig. 4b), in line with their described elevated magnitudes in studies of pediatric and adult progressors [13–15].

(a,b) Representative FACS staining and frequency of CD107a+ and IFN-γ+ CD8^+^ T cell upon stimulation with Gag, Env, and Nef in pediatric elite controller (PEC), pediatric non-progressor (PNP) and pediatric progressor (PP). (c) Functional profile of Gag-specific CD8^+^ T cells of each group. Each slice of the pie chart represents the average relative proportions of each possible combination of the functions measured. Slices are colored according to the number of simultaneous functions. The arcs illustrate the specific cytokine response.

**Fig. 5:**
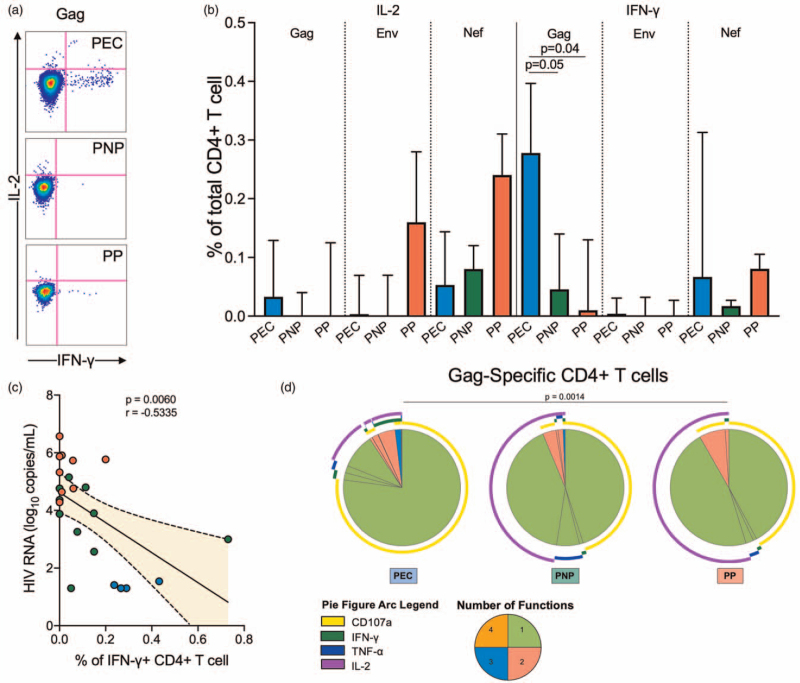
HIV-specific response of CD4^+^ T cells.

Gag-specific CD4^+^ T-cell responses differed considerably in magnitude and quality from the CD8^+^ T-cell responses. These cells expressed more IFN-γ upon Gag stimulation in PECs (Fig. 5a and b) and the frequency of IFN-γ+ in CD4^+^ T cells negatively correlated with plasma HIV-RNA copies (Fig. 5c), consistent with previous data [16]. The CD4^+^ T cells that responded to Gag pool were more polyfunctional in the PECs compared with pediatric progressors (*P* = 0.0014, Fig. 5d).

(a,b) Representative FACS staining and frequency of IL-2+ and IFN-γ+ CD4^+^ T cell upon stimulation with Gag, Env, and Nef in pediatric elite controller (PEC), pediatric non-progressor (PNP) and pediatric progressor (PP). (c). Correlation between viral load and percentage of CD4^+^ T cell secreting IFN-γ upon Gag pool stimulation for each individual. Spearman correlation and Best-fit line are shown. (d) Functional profile of Gag-specific CD4^+^ T cells of each group. Each slice of the pie chart represents the average relative proportions of each possible combination of the functions measured. Slices are colored according to the number of simultaneous functions. The arcs illustrate the specific cytokine response.

## Discussion

Viremic control in the pediatric population is rare and the effector mechanisms underlying the suppression of viremia are unclear. Despite only a small number of PECs individuals available to study and the cautions we need to have when interpreting statistical differences here, it was possible to identify features in the T-cell compartment and in the antiviral response that distinguish PECs from ART-naive HIV-infected children/adolescents who also maintain normal-for-age CD4^+^ T-cell counts but do not control viremia (PNPs). The principal differences are in a more polyfunctional Gag-specific CD8^+^ T-cell responses combined with higher frequencies of Gag-specific CD4^+^ T-cell IFN-γ responses in PECs compared with PNPs. These data provide some clues as to how HIV-specific T-cell responses in PECs are distinct from those in pediatric viremic nonprogressors.

In ART-naive HIV-infected children, control of viral replication is a gradual and usually incomplete process that takes several years, which in adults is completed within weeks of infection [4,6]. In our previous studies, we have shown slow decline of viraemia over the first 5 years of life among ART-naive PNPs who maintain normal-for-age CD4^+^ T-cell counts [3], and also, we have shown that viremic control in PECs is only achieved after a median of 6.5 years of infection [4]. By contrast, viral setpoint in adults is achieved within 4 weeks of infection [17], and elite control achieved within 12 months at most [4,7]. This difference by which elite control is reached in children and adults can represent distinct strategies to achieve viremic control and reflects the contrasting developmental stages of the immune system. In adults, the speed and extent of activation of CD8^+^ T cells following infection predicts the degree to which viremia will be suppressed [18]. Rapid immune control of viremia requires high levels of immune activation of T cells, but in early life, these high levels of immune activation result in accelerated CD4^+^ T-cells decline [3]. Like PNPs, PECs have an immunophenotype resembling that of the natural hosts of nonpathogenic SIV infection, the hallmark being a low degree of immune activation, lack of exhaustion of the CD4^+^ T-cell population, and the low expression of CCR5 in long-lived memory cells [2,3,19]. This immunophenotype appears to be more compatible with the tolerogenic immune environment in early life HIV [2,3,20] than in adult infection, wherein viremic nonprogressors are very rare [19,21]. In the current study, this phenotype of low immune activation and restriction of higher CCR5 expression to short-lived CD4^+^ T-cell subsets is even more marked in the PECs.

As children transition toward adulthood through adolescence, the immune response progressively shifts toward a more aggressive adult-like antiviral response that is eventually associated with acquisition of viral control in PEC [4,20]. Whereas in adults, 60% of white elite controllers express one of HLA-B∗57 or HLA-B∗27 [11], and in African elite controllers, 60% express one or more of HLA-B∗57, HLA-B∗58 : 01, or HLA-B∗81 : 01 [9,11,12,22], only two out of seven HLA class I typed PECs expressed a protective molecule and consistent with previous pediatric data [23]. However, genetic associations explain only 25% of host control in adults and other features also play a role in the antiviral response [9]. The polyfunctionality of the HIV-specific CD8^+^ T-cell response has been shown to be enhanced in adults elite controllers compared with normal progressors [24–28]; however, the rarity of adult viremic nonprogressors has made it difficult to determine whether CD8^+^ T-cell polyfunctionality is a cause or consequence of maintained CD4^+^ T-cell counts among adult elite controllers. The existence of PNPs who have maintained normal-for-age CD4^+^ T-cell counts and yet have markedly reduced HIV-specific CD8^+^ T-cell polyfunctionality compared with PECs suggests that CD8^+^ T-cell polyfunctionality can be an important driver of PECs.

A further marker observed here distinguishing these groups was expression of the exhaustion markers PD-1 and CD39. We noted a distinct upregulation of CD39 and downregulation of CD73 in pediatric progressors and PNPs but not PECs when compared with HEUs, which was especially marked in the CD8^+^ T-cells. In adults, CD39 is positively correlated with disease progression and exhaustion, while the CD73 exert the opposite effect [29,30]. CD39 is an NTPDase classically upregulated in activated cells during inflammation [31]. By contrast, CD73, which functions to convert adenosine monophosphate into adenosine, is downregulated in CD8^+^ T cells upon activation and in terminally differentiated cells [31]. Indeed, the adenosine produced by CD73 is important in the generation and maintenance of memory cells [32]. This phenotype, together with a low expression of CD38, HLA-DR, and PD-1 in the CD8^+^ T-cell compartment in PECs, maintains a more effective and sustained effector function.

We also observed an increased IFN-γ frequency on Gag-specific CD4^+^ T-cell in PECs associated with a more polyfunctional phenotype compared with pediatric progressors. Gag-specific CD4^+^ T-cell responses have been associated with a lower viral set point in adults and children, although not always in association with high expression of IFN-γ [3,16,30,33]. As noted in previous studies of both adult and pediatric infection [3,15,34], we observed here again the association of Nef-specific CD8^+^ T-cell responses with ineffective control of viraemia. Together, these data support the notion that Gag-specific CD4^+^ and CD8^+^ T-cell activity contribute increasingly through childhood to control of viremia, and may play a progressively more important part in antiviral immunity as part of strategies to achieve functional cure in children from mid-childhood onwards.

As observed in adult viremic long-term nonprogressors [35,36] and elite controllers [37–39], the total HIV-DNA in ART-naive children was lower in the PECs than in the pediatric viremic groups. Their ability to control the viral reservoir could be explained by their ability to control viral replication or to resist viral infection. The CD4^+^ T-cell compartment of PECs is mainly populated with naive and central memory cells with the lowest level of immune activation and exhaustion among the individuals living with HIV. In previous studies in PNPs, total HIV-DNA were mainly integrated in the short-lived effector memory CD4^+^ T cells, in contrast to higher copy numbers in the long-lived central memory in future adult progressors [3]. In adult studies, low levels of HIV-DNA viral reservoir have also been related to lower immune activation in both CD4^+^ and CD8^+^ T-cells [40–42]. However, in adults achieving low viral load is related to the ability of the HIV-specific CD8^+^ T-cell response in acute infection to become rapidly and fully activated [18]. The low immune activation observed in association with chronic elite control in adults therefore is the consequence of the aggressive response in acute infection. In PECs, by contrast, low immune activation presumably precedes immune control of viraemia.

It is important to acknowledge limitations to our study that include its cross-sectional nature and the low number of PECs that could be assessed. The rarity of the elite control phenotype, combined with the introduction of the universal treatment guidelines, make the generated data unique, even if somewhat anecdotal. However, despite low the numbers, we were able to identify clear-cut differences between PECs and other pediatric phenotypes, shedding light on the immune strategies that have been adopted to achieve viral suppression in the context of vertical transmission. The small sample size also does not allow us to exclude a contributing role for protective HLAs in pediatric elite control. We also understand that HEUs, the control group, are immunologically different from HIV-unexposed uninfected children (HUUs), but they are still distinct from children living with HIV and are helpful as a reference group. They are also likely to match the HIV-infected groups better socioeconomically than HUUs.

We have previously determined that 100% of more than 600 HIV-infected individuals from Durban, South Africa are C clade infected [43]. Therefore, it is reasonable to assume that the PNPs and pediatric progressors and PEC-4, all from Durban, were also C clade infected. However, because viral loads were so low in the PEC individuals, we were unable to determine the clade of infection in these PECs. It is possible that some of the PECs were infected by non-C clade viruses, in particular PEC-1 and PEC-2, whose country of origin were Ghana and Nigeria, respectively. We only measured total HIV-1 DNA, which limits our capacity to interpret the viral reservoir in these populations further. Finally, innate immunity and B-cells were not assessed in this study, and these cells are likely also to contribute to viremic control. Previous studies have demonstrated a correlation between viral load and the neutralization breadth of anti-HIV antibodies [3,44]. In addition, natural killer (NK) cells exert important effector functions in adult infection and achieve full maturity in children much earlier than T cells [45,46] and the role of these cells is the focus of ongoing work.

In summary, we have demonstrated that PECs have a T-cell compartment with very low levels of immune activation and exhaustion that is close to those observed in HEUs and shares similar features with the SIV nonpathogenetic infection. However, viral suppression is associated with a robust HIV-specific T-cell response, similar to the adult elite controllers. As demonstrated previously, low viraemia in pediatric infection is associated with high-frequency Gag-specific CD4^+^ T-cell but not high-frequency CD8^+^ T-cell responses. The presence of a CD8^+^ T-cell compartment that is not only populated with a more robust HIV-specific activity but also less driven to exhaustion and cell death, distinguishes the PECs from pediatric viremic noncontrollers. This dual phenotype can provide important clues to remission strategies in posttreatment controllers, wherein viremic suppression is typically achieved in absence of protective HLA molecules [47].

## Acknowledgements

This work was fully supported by the Wellcome Trust [PG WTIA Grant WT104748MA], the National Institutes of Health [PG RO1-AI133673], and partially with federal funds from the Frederick National Laboratory for Cancer Research [Contract No. HHSN261200800001E]. The content of this publication does not necessarily reflect the views or policies of the Department of Health and Human Services, nor does mention of trade names, commercial products, or organizations imply endorsement by the US Government. This Research was supported in part by the Intramural Research Program of the NIH, Frederick National Lab, Center for Cancer Research.

V.A.V. and P.G. wrote the article, contributed to the study conception and design, and contributed to the acquisition, analysis, and interpretation of the data; E.A., J.M., J.R., C.F.G., D.P., B.T., M.P.M., and M.C.G. contributed to the acquisition of the data; M.M., C.B., J.M.P., A.B., M.C., G.T.W., and J.F. contributed with acquisition and interpretation of the data.

### Conflicts of interest

None declared.

## Supplementary Material

Supplemental Digital Content

## References

[1] Paul ME Mao C Charurat M Serchuck L Foca M Hayani K. Predictors of immunologic long-term nonprogression in HIV-infected children: implications for initiating therapy. *J Allergy Clin Immunol* 2005; 115:848–855. 15806009 10.1016/j.jaci.2004.11.054

[2] Silvestri G Sodora DL Koup RA Paiardini M O’Neil SP McClure HM. Nonpathogenic SIV infection of sooty mangabeys is characterized by limited bystander immunopathology despite chronic high-level viremia. *Immunity* 2003; 18:441–452. 12648460 10.1016/s1074-7613(03)00060-8

[3] Muenchhoff M Adland E Karimanzira O Crowther C Pace M Csala A. Nonprogressing HIV-infected children share fundamental immunological features of nonpathogenic SIV infection. *Sci Transl Med* 2016; 8:358ra125. 10.1126/scitranslmed.aag1048 PMC6087524 27683550

[4] Vieira VA Zuidewind P Muenchhoff M Roider J Millar J Clapson M. Strong sex bias in elite control of paediatric HIV infection. *AIDS* 2019; 33:67–75. 30325765 10.1097/QAD.0000000000002043 PMC6750143

[5] Deeks SG Walker BD. Human immunodeficiency virus controllers: mechanisms of durable virus control in the absence of antiretroviral therapy. *Immunity* 2007; 27:406–416. 17892849 10.1016/j.immuni.2007.08.010

[6] Yang OO Cumberland WG Escobar R Liao D Chew KW. Demographics and natural history of HIV-1-infected spontaneous controllers of viremia. *AIDS* 2017; 31:1091–1098. 28301422 10.1097/QAD.0000000000001443 PMC5657480

[7] Madec Y Boufassa F Porter K Prins M Sabin C d’Arminio Monforte A. Natural history of HIV-control since seroconversion. *AIDS* 2013; 27:2451–2460. 23912979 10.1097/01.aids.0000431945.72365.01

[8] Morón-López S Puertas MC Gálvez C Navarro J Carrasco A Esteve M. Sensitive quantification of the HIV-1 reservoir in gut-associated lymphoid tissue. *PLoS One* 2017; 12:e0175899. 28414780 10.1371/journal.pone.0175899 PMC5393620

[9] Pereyra F Jia X McLaren PJ Telenti A de Bakker PI Walker BD. The major genetic determinants of HIV-1 control affect HLA class I peptide presentation. *Science* 2010; 330:1551–1557. 21051598 10.1126/science.1195271 PMC3235490

[10] Miura T Brockman MA Schneidewind A Lobritz M Pereyra F Rathod A. HLA-B57/B∗5801 human immunodeficiency virus type 1 elite controllers select for rare gag variants associated with reduced viral replication capacity and strong cytotoxic T-lymphocyte [corrected] recognition. *J Virol* 2009; 83:2743–2755. 19116253 10.1128/JVI.02265-08 PMC2648254

[11] Pereyra F Addo MM Kaufmann DE Liu Y Miura T Rathod A. Genetic and immunologic heterogeneity among persons who control HIV infection in the absence of therapy. *J Infect Dis* 2008; 197:563–571. 18275276 10.1086/526786

[12] Leslie A Matthews PC Listgarten J Carlson JM Kadie C Ndung’u T. Additive contribution of HLA class I alleles in the immune control of HIV-1 infection. *J Virol* 2010; 84:9879–9888. 20660184 10.1128/JVI.00320-10 PMC2937780

[13] Sunshine J Kim M Carlson JM Heckerman D Czartoski J Migueles SA. Increased sequence coverage through combined targeting of variant and conserved epitopes correlates with control of HIV replication. *J Virol* 2014; 88:1354–1365. 24227851 10.1128/JVI.02361-13 PMC3911649

[14] Edwards BH Bansal A Sabbaj S Bakari J Mulligan MJ Goepfert PA. Magnitude of functional CD8+ T-cell responses to the gag protein of human immunodeficiency virus type 1 correlates inversely with viral load in plasma. *J Virol* 2002; 76:2298–2305. 11836408 10.1128/jvi.76.5.2298-2305.2002 PMC135950

[15] Kiepiela P Ngumbela K Thobakgale C Ramduth D Honeyborne I Moodley E. CD8+ T-cell responses to different HIV proteins have discordant associations with viral load. *Nat Med* 2007; 13:46–53. 17173051 10.1038/nm1520

[16] Prendergast A Goodliffe H Clapson M Cross R Tudor-Williams G Riddell A. Gag-specific CD4+ T-cell responses are associated with virological control of paediatric HIV-1 infection. *AIDS* 2011; 25:1329–1331. 21505296 10.1097/QAD.0b013e3283478575

[17] Robb ML Eller LA Kibuuka H Rono K Maganga L Nitayaphan S. Prospective study of acute HIV-1 infection in adults in East Africa and Thailand. *N Engl J Med* 2016; 374:2120–2130. 27192360 10.1056/NEJMoa1508952 PMC5111628

[18] Ndhlovu ZM Kamya P Mewalal N Kløverpris HN Nkosi T Pretorius K. Magnitude and kinetics of CD8+ T cell activation during hyperacute HIV infection impact viral set point. *Immunity* 2015; 43:591–604. 26362266 10.1016/j.immuni.2015.08.012 PMC4575777

[19] Klatt NR Bosinger SE Peck M Richert-Spuhler LE Heigele A Gile JP. Limited HIV infection of central memory and stem cell memory CD4+ T cells is associated with lack of progression in viremic individuals. *PLoS Pathog* 2014; 10:e1004345. 25167059 10.1371/journal.ppat.1004345 PMC4148445

[20] Muenchhoff M Prendergast AJ Goulder PJ. Immunity to HIV in early life. *Front Immunol* 2014; 5:391. 25161656 10.3389/fimmu.2014.00391 PMC4130105

[21] Rotger M Dalmau J Rauch A McLaren P Bosinger SE Martinez R. Comparative transcriptomics of extreme phenotypes of human HIV-1 infection and SIV infection in sooty mangabey and rhesus macaque. *J Clin Invest* 2011; 121:2391–2400. 21555857 10.1172/JCI45235 PMC3104754

[22] Carlson JM Listgarten J Pfeifer N Tan V Kadie C Walker BD. Widespread impact of HLA restriction on immune control and escape pathways of HIV-1. *J Virol* 2012; 86:5230–5243. 22379086 10.1128/JVI.06728-11 PMC3347390

[23] Adland E Paioni P Thobakgale C Laker L Mori L Muenchhoff M. Discordant impact of HLA on viral replicative capacity and disease progression in pediatric and adult HIV infection. *PLoS Pathog* 2015; 11:e1004954. 26076345 10.1371/journal.ppat.1004954 PMC4468173

[24] Betts MR Nason MC West SM De Rosa SC Migueles SA Abraham J. HIV nonprogressors preferentially maintain highly functional HIV-specific CD8+ T cells. *Blood* 2006; 107:4781–4789. 16467198 10.1182/blood-2005-12-4818 PMC1895811

[25] Migueles SA Laborico AC Shupert WL Sabbaghian MS Rabin R Hallahan CW. HIV-specific CD8+ T cell proliferation is coupled to perforin expression and is maintained in nonprogressors. *Nat Immunol* 2002; 3:1061–1068. 12368910 10.1038/ni845

[26] Migueles SA Mendoza D Zimmerman MG Martins KM Toulmin SA Kelly EP. CD8(+) T-cell cytotoxic capacity associated with human immunodeficiency virus-1 control can be mediated through various epitopes and human leukocyte antigen types. *EBioMedicine* 2015; 2:46–58. 26137533 10.1016/j.ebiom.2014.12.009 PMC4485486

[27] Sáez-Cirión A Lacabaratz C Lambotte O Versmisse P Urrutia A Boufassa F. HIV controllers exhibit potent CD8 T cell capacity to suppress HIV infection ex vivo and peculiar cytotoxic T lymphocyte activation phenotype. *Proc Natl Acad Sci U S A* 2007; 104:6776–6781. 17428922 10.1073/pnas.0611244104 PMC1851664

[28] Almeida JR Price DA Papagno L Arkoub ZA Sauce D Bornstein E. Superior control of HIV-1 replication by CD8+ T cells is reflected by their avidity, polyfunctionality, and clonal turnover. *J Exp Med* 2007; 204:2473–2485. 17893201 10.1084/jem.20070784 PMC2118466

[29] Tóth I Le AQ Hartjen P Thomssen A Matzat V Lehmann C. Decreased frequency of CD73+CD8+ T cells of HIV-infected patients correlates with immune activation and T cell exhaustion. *J Leukoc Biol* 2013; 94:551–561. 23709688 10.1189/jlb.0113018

[30] Gaiha GD McKim KJ Woods M Pertel T Rohrbach J Barteneva N. Dysfunctional HIV-specific CD8+ T cell proliferation is associated with increased caspase-8 activity and mediated by necroptosis. *Immunity* 2014; 41:1001–1012. 25526311 10.1016/j.immuni.2014.12.011 PMC4312487

[31] Allard B Longhi MS Robson SC Stagg J. The ectonucleotidases CD39 and CD73: novel checkpoint inhibitor targets. *Immunol Rev* 2017; 276:121–144. 28258700 10.1111/imr.12528 PMC5338647

[32] Bono MR Fernández D Flores-Santibáñez F Rosemblatt M Sauma D. CD73 and CD39 ectonucleotidases in T cell differentiation: beyond immunosuppression. *FEBS Lett* 2015; 589:3454–3460. 26226423 10.1016/j.febslet.2015.07.027

[33] Adland E Mori L Laker L Csala A Muenchhoff M Swordy A. Recovery of effective HIV-specific CD4+ T-cell activity following antiretroviral therapy in paediatric infection requires sustained suppression of viraemia. *AIDS* 2018; 32:1413–1422. 29734220 10.1097/QAD.0000000000001844 PMC6039399

[34] Leitman EM Thobakgale CF Adland E Ansari MA Raghwani J Prendergast AJ. Role of HIV-specific CD8. *J Exp Med* 2017; 214:3239–3261. 28983013 10.1084/jem.20162123 PMC5679167

[35] Lambotte O Boufassa F Madec Y Nguyen A Goujard C Meyer L. HIV controllers: a homogeneous group of HIV-1-infected patients with spontaneous control of viral replication. *Clin Infect Dis* 2005; 41:1053–1056. 16142675 10.1086/433188

[36] Garbuglia AR Salvi R Di Caro A Montella F Di Sora F Recchia O. Peripheral lymphocytes of clinically nonprogressor patients harbor inactive and uninducible HIV proviruses. *J Med Virol* 1995; 46:116–121. 7636497 10.1002/jmv.1890460206

[37] Graf EH Mexas AM Yu JJ Shaheen F Liszewski MK Di Mascio M. Elite suppressors harbor low levels of integrated HIV DNA and high levels of 2-LTR circular HIV DNA compared to HIV+ patients on and off HAART. *PLoS Pathog* 2011; 7:e1001300. 21383972 10.1371/journal.ppat.1001300 PMC3044690

[38] de Masson A Kirilovsky A Zoorob R Avettand-Fenoel V Morin V Oudin A. Blimp-1 overexpression is associated with low HIV-1 reservoir and transcription levels in central memory CD4+ T cells from elite controllers. *AIDS* 2014; 28:1567–1577. 24804861 10.1097/QAD.0000000000000295

[39] García M Górgolas M Cabello A Estrada V Ligos JM Fernández-Guerrero M. Peripheral T follicular helper cells make a difference in HIV reservoir size between elite controllers and patients on successful cART. *Sci Rep* 2017; 7:16799. 29196729 10.1038/s41598-017-17057-y PMC5711909

[40] Khoury G Fromentin R Solomon A Hartogensis W Killian M Hoh R. Human immunodeficiency virus persistence and T-cell activation in blood, rectal, and lymph node tissue in human immunodeficiency virus-infected individuals receiving suppressive antiretroviral therapy. *J Infect Dis* 2017; 215:911–919. 28453847 10.1093/infdis/jix039 PMC5407052

[41] Cockerham LR Siliciano JD Sinclair E O’Doherty U Palmer S Yukl SA. CD4+ and CD8+ T cell activation are associated with HIV DNA in resting CD4+ T cells. *PLoS One* 2014; 9:e110731. 25340755 10.1371/journal.pone.0110731 PMC4207702

[42] Yukl SA Gianella S Sinclair E Epling L Li Q Duan L. Differences in HIV burden and immune activation within the gut of HIV-positive patients receiving suppressive antiretroviral therapy. *J Infect Dis* 2010; 202:1553–1561. 20939732 10.1086/656722 PMC2997806

[43] Kawashima Y Pfafferott K Frater J Matthews P Payne R Addo M. Adaptation of HIV-1 to human leukocyte antigen class I. *Nature* 2009; 458:641–645. 19242411 10.1038/nature07746 PMC3148020

[44] Goo L Chohan V Nduati R Overbaugh J. Early development of broadly neutralizing antibodies in HIV-1-infected infants. *Nat Med* 2014; 20:655–658. 24859529 10.1038/nm.3565 PMC4060046

[45] Alter G Teigen N Ahern R Streeck H Meier A Rosenberg ES. Evolution of innate and adaptive effector cell functions during acute HIV-1 infection. *J Infect Dis* 2007; 195:1452–1460. 17436225 10.1086/513878

[46] Yabuhara A Kawai H Komiyama A. Development of natural killer cytotoxicity during childhood: marked increases in number of natural killer cells with adequate cytotoxic abilities during infancy to early childhood. *Pediatr Res* 1990; 28:316–322. 1700360 10.1203/00006450-199010000-00002

[47] Sáez-Cirión A Bacchus C Hocqueloux L Avettand-Fenoel V Girault I Lecuroux C. Posttreatment HIV-1 controllers with a long-term virological remission after the interruption of early initiated antiretroviral therapy ANRS VISCONTI Study. *PLoS Pathog* 2013; 9:e1003211. 23516360 10.1371/journal.ppat.1003211 PMC3597518

