# Supplementary material for: Robust HIV-specific CD4+ and CD8+ T-cell responses distinguish elite control in adolescents living with HIV from viremic nonprogressors
Source: AIDS. 2021 Oct 1;36(1):95–105. doi: 10.1097/QAD.0000000000003078 (PMC8654249; doi:10.1097/QAD.0000000000003078)
Supplement: Supplemental Digital Content [file aids-36-095-s001.docx]

**
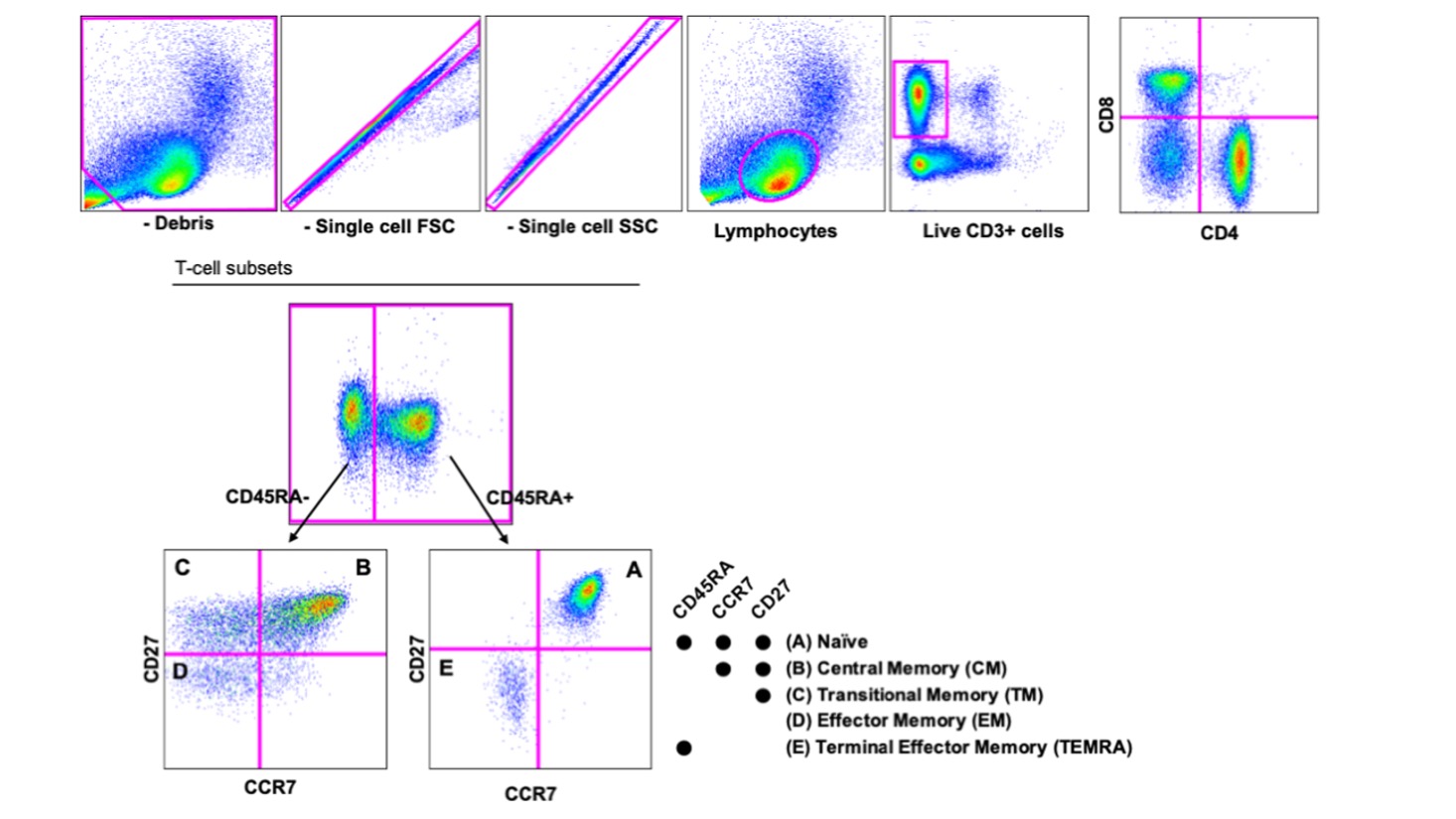
**

**Supplemental Figure 1. Gating strategy for surface immunophenotype.** Scheme to gate the naïve and memory subsets of live CD8+ and CD4+ T-cells. The gate position for the other markers was based on the Fluorescence Minus One (FMO) control.

**
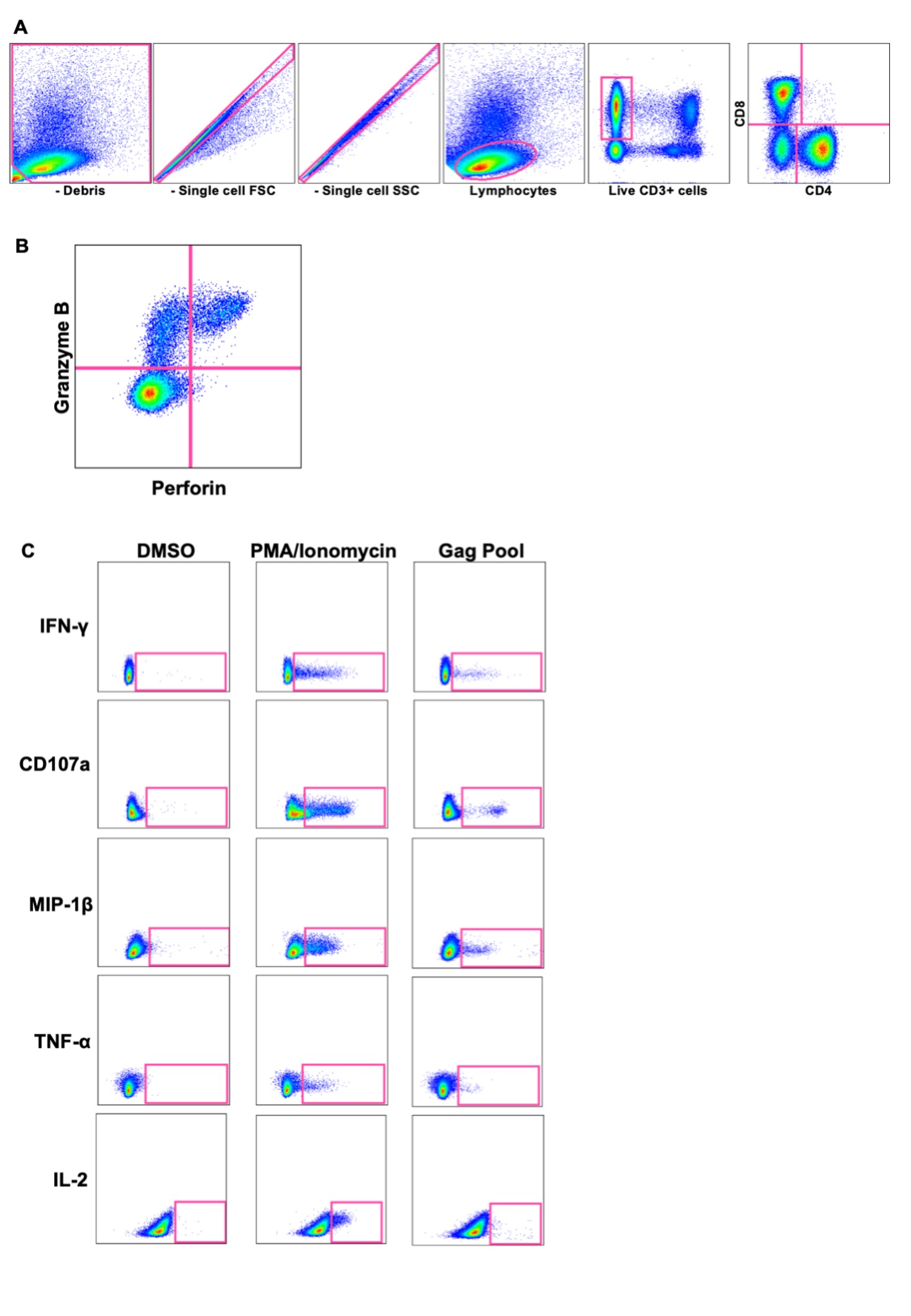
**

**Supplemental Figure 2. Gating strategy for intracellular cytokine staining.** A. Scheme to gate CD4+ and CD8+ T-cells. B. Typical plot for Granzyme B and Perforin. C. Typical DMSO, PMA/Ionomycin and Gag pool plots for IFN-γ, CD107a, MIP-1β, TNF-α and IL-2.

**Supplemental Figure 3.** **Longitudinal clinical data of the additional PECs with typed HLA-I.** Longitudinal plasma HIV-RNA (red triangles) and absolute CD4+ T-cell count (blue circles) of PEC-5, PEC-6 and PEC-7. The horizontal dashed line represents the plasma HIV-RNA of 50 copies/mL. The 10th, 50th and 90th percentiles for absolute and percentage CD4+ T-cell for HIV-uninfected children are represented by the three grey lines.

**Supplemental Table 1.** Individual clinical data

|  |  |  | **Timepoint** | | | | | | |
| --- | --- | --- | --- | --- | --- | --- | --- | --- | --- |
| **Group** | **Sex** | **Country of origin** | **Age (years)** | **Plasma HIV-RNA (copies/mL)** | **Total HIV-DNA (copies/10^6^ CD4+ T-cells)** | **Absolute CD4+ T-cell count (cells/mm^3^)** | **Relative CD4+ T-cell (%)** | **CD4:CD8 ratio** |  |
| PNP-01 | Male | South Africa | 16.4 | 1,800 | 2,267 | 779 | 34 | 0.42 |  |
| PNP-02 | Female | South Africa | 16.9 | 20 | 4,885 | 621 | 34 | 1.75 |  |
| PNP-03 | Male | South Africa | 14.1 | 1,700 | 647 | 982 | 33 | 1.63 |  |
| PNP-04 | Female | South Africa | 17.9 | 1,000 | 238 | 1007 | 34 | 1.37 |  |
| PNP-05 | Male | South Africa | 11.3 | 1,500 | 7,293 | 819 | 38 | 1.67 |  |
| PNP-06 | Female | South Africa | 10.1 | 1,800 | 11,118 | 933 | 45 | 1.69 |  |
| PNP-07 | Female | South Africa | 11.1 | 140,000 | 15,445 | 629 | 38 | 1.24 |  |
| PNP-08 | Male | South Africa | 12.7 | 8,000 | 11,781 | 463 | 31 | 0.96 |  |
| PNP-09 | Female | South Africa | 13.0 | 570 | 12,981 | 547 | 22 | 0.52 |  |
| PNP-10 | Male | South Africa | 14.2 | 370 | 40,435 | 550 | 28 | 0.45 |  |
| PNP-11 | Male | South Africa | 11.9 | 23,000 | 22,370 | 721 | 28 | 0.81 |  |
| PNP-12 | Female | South Africa | 15.2 | 59,000 | 6,274 | 1345 | 25 | 0.53 |  |
| PNP-13 | Male | South Africa | 10.4 | 23,000 | 7,120 | 954 | 34 | 0.89 |  |
| PP-01 | Female | South Africa | 13.2 | 470,000 | 11,576 | 387 | 18 | 0.43 |  |
| PP-02 | Male | South Africa | 14.0 | 44,000 | 86,633 | 164 | 7 | 0.16 |  |
| PP-03 | Female | South Africa | 12.7 | 19,000 | 79,728 | 86 | 7 | 0.10 |  |
| PP-04 | Male | South Africa | 17.3 | 210,000 | 142,913 | 476 | 9 | 0.09 |  |
| PP-05 | Male | South Africa | 18.1 | 8,200,000 | 6,260 | 375 | 18 | 0.36 |  |
| PP-06 | Male | South Africa | 15.2 | 5,400,000 | 558 | 235 | 21 | 0.34 |  |
| PP-07 | Female | South Africa | 11.5 | 7,400,000 | 25,594 | 251 | 9 | 0.28 |  |
| PP-08 | Female | South Africa | 10.7 | 5,900,000 | 80,950 | 242 | 9 | 0.12 |  |
| PP-09 | Male | South Africa | 14.9 | 57,000 | 19,554 | 41 | 3 | 0.08 |  |
| PP-10 | Female | South Africa | 12.5 | 3,700,000 | 14,386 | 321 | 8 | 0.36 |  |
| HEU-01 | Male | South Africa | 12.3 | - | - | - | - | - |  |
| HEU-02 | Female | South Africa | 16.7 | - | - | - | - | - |  |
| HEU-03 | Male | South Africa | 15 | - | - | - | - | - |  |
| HEU-04 | Female | South Africa | 12 | - | - | - | - | - |  |
| HEU-05 | Male | South Africa | 14 | - | - | - | - | - |  |
| HEU-06 | Female | South Africa | 10.2 | - | - | - | - | - |  |
| HEU-07 | Female | South Africa | 14.5 | - | - | - | - | - |  |
| HEU-08 | Female | South Africa | 16.1 | - | - | - | - | - |  |
| HEU-09 | Male | South Africa | 18.9 | - | - | - | - | - |  |

**
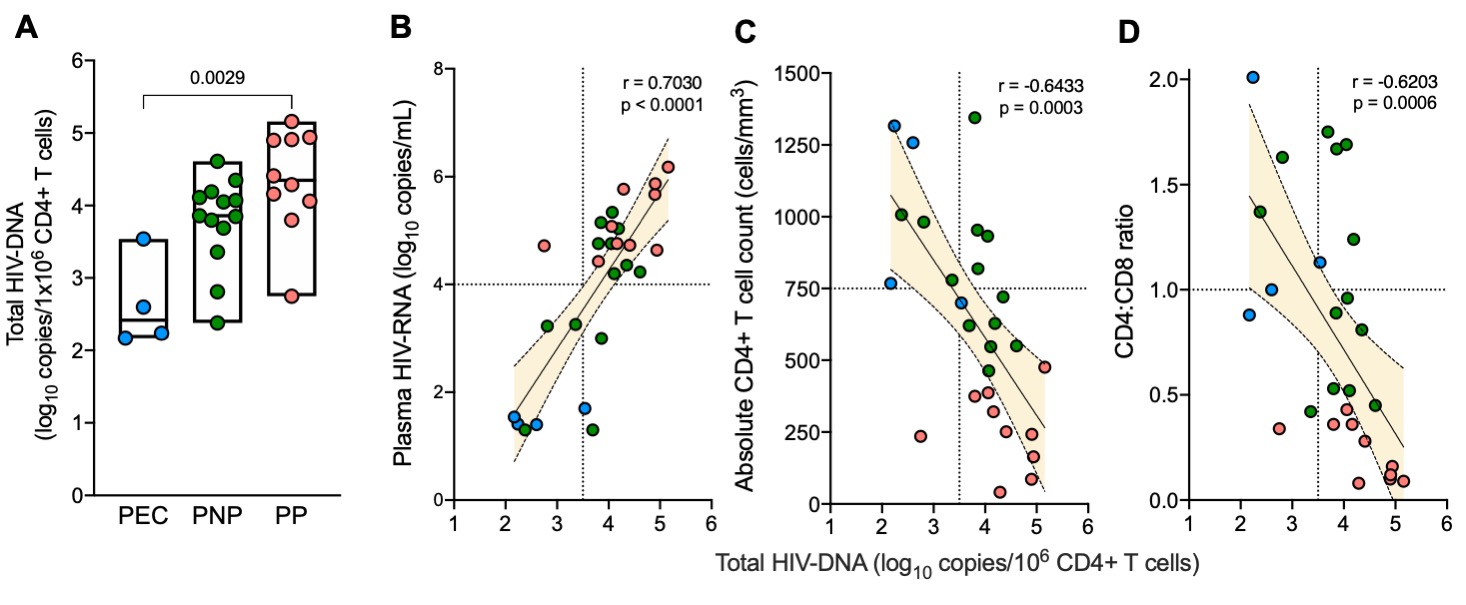
**

**Supplemental Figure 4. Total HIV-DNA levels distinguish PEC from PP.** A. Total HIV-DNA copies adjusted to CD4+ T-cell count are Paediatric Elite Controllers (PECs, blue), Paediatric Non-Progressors (PNPs, green), Paediatric Progressors (PPs, red). Boxplots represent median values and minimum and maximum values. B-D Plasma HIV-RNA (B), absolute CD4+ T-cell count (C), and CD4:CD8 ratio (D) correlated with total HID-DNA levels adjusted by CD4+ T-cell. Statistical comparison between groups was done with Kruskal-Wallis test followed by Dunn’s test for multiple comparisons. Spearman rank tests were used for correlations. Best-fit line and 95% confidence bands are shown.

**Supplemental Table 2. Immunophenotype of CD4+ T-cells.** Comparison was done with Kruskal-Wallis’ test followed by Dunn’s test to correct for multiple comparisons.

|  | **p-value** | | | | | | | | | | | | |
| --- | --- | --- | --- | --- | --- | --- | --- | --- | --- | --- | --- | --- | --- |
| **CD4+ T-cell** | ***HEU vs. PEC*** | ***HEU vs. PNP*** | ***HEU vs. PP*** | | ***PEC vs. PNP*** | | ***PEC vs. PP*** | | ***PNP vs. PP*** | | |  |  |
| **Total** |  |  | |  | |  | |  | |  | |  |  |
| CCR5+ | - | - | | - | | - | | - | | - | |  |  |
| HLA-DR+CD38+ | - | - | | 0.0007 | | - | | - | | - | |  |  |
| PD-1+ | - | - | | 0.0294 | | - | | - | | - | |  |  |
| CD39+ | - | - | | 0.0096 | | - | | - | | - | |  |  |
| CD73+ | - | - | | - | | - | | - | | - | |  |  |
| **Naïve** |  |  | |  | |  | |  | |  | |  |  |
| CCR5+ | - | - | | - | | - | | - | | | - | |  |
| HLA-DR+CD38+ | - | - | | - | | - | | - | | | - | |  |
| PD-1+ | - | - | | - | | - | | - | | | - | |  |
| CD39+ | - | - | | - | | - | | - | | | - | |  |
| CD73+ | - | - | | 0.0074 | | - | | - | | | - | |  |
| **CM** |  |  | |  | |  | |  | | |  | |  |
| CCR5+ | - | - | | - | | - | | - | | | - | |  |
| HLA-DR+CD38+ | - | 0.0409 | | 0.0003 | | - | | 0.0033 | | | - | |  |
| PD-1+ | - | - | | 0.0017 | | - | | - | | | - | |  |
| CD39+ | - | - | | 0.0039 | | - | | - | | | - | |  |
| CD73+ | - | - | | - | | - | | - | | | - | |  |
| **TM** |  |  | |  | |  | |  | | |  | |  |
| CCR5+ | - | - | | 0.0015 | | - | | 0.024 | | | - | |  |
| HLA-DR+CD38+ | - | - | | 0.0035 | | - | | 0.0024 | | | - | |  |
| PD-1+ | - | - | | 0.0019 | | - | | - | | | 0.0333 | |  |
| CD39+ | - | - | | 0.0390 | | - | | 0.0129 | | | - | |  |
| CD73+ | - | - | | - | | - | | - | | | - | |  |
| **EM** |  |  | |  | |  | |  | | |  | |  |
| CCR5+ | - | - | | - | | - | | - | | | - | |  |
| HLA-DR+CD38+ | - | 0.0331 | | 0.0008 | | - | | - | | | - | |  |
| PD-1+ | - | - | | - | | - | | - | | | - | |  |
| CD39+ | - | - | | - | | - | | - | | | - | |  |
| CD73+ | - | - | | - | | - | | - | | | - | |  |

**Supplemental Table 3. Immunophenotype of CD8+ T-cells.** Comparison was done with Kruskal-Wallis’ test followed by Dunn’s test to correct for multiple comparisons.

|  | **p-value** | | | | | | |
| --- | --- | --- | --- | --- | --- | --- | --- |
| **CD8+ T-cell** | ***HEU vs. PEC*** | ***HEU vs. PNP*** | ***HEU vs. PP*** | ***PEC vs. PNP*** | ***PEC vs. PP*** | ***PNP vs. PP*** |  |
| **Total** |  |  |  |  |  |  |  |
| CCR5+ | - | - | - | - | - | - |  |
| HLA-DR+CD38+ | - | 0.0312 | 0.0013 | - | 0.0503 | - |  |
| PD-1+ | - | - | 0.0002 | - | - | - |  |
| CD39+ | - | 0.023 | 0.0004 | - | - | - |  |
| CD73+ | - | - | 0.0019 | - | - | 0.0282 |  |
| **Naïve** |  |  |  |  |  |  |  |
| CCR5+ | - | - | - | - | - | - |  |
| HLA-DR+CD38+ | - | - | 0.0105 | - | - | - |  |
| PD-1+ | - | - | - | - | - | - |  |
| CD39+ | - | - | 0.0002 | - | 0.0084 | - |  |
| CD73+ | - | - | - | - | - | - |  |
| **CM** |  |  |  |  |  |  |  |
| CCR5+ | - | - | - | - | - | - |  |
| HLA-DR+CD38+ | - | - | 0.0005 | - | - | - |  |
| PD-1+ | - | 0.0111 | 0.0016 | - | - | - |  |
| CD39+ | - | - | 0.001 | - | - | - |  |
| CD73+ | - | - | - | - | - | - |  |
| **TM** |  |  |  |  |  |  |  |
| CCR5+ | - | - | 0.0103 | - | - | - |  |
| HLA-DR+CD38+ | - | 0.0415 | 0.0065 | - | - | - |  |
| PD-1+ | - | - | 0.0003 | - | 0.0045 | - |  |
| CD39+ | - | 0.0148 | 0.0038 | - | - | - |  |
| CD73+ | - | - | 0.0053 | - | - | - |  |
| **EM** |  |  |  |  |  |  |  |
| CCR5+ | - | - | - | - | - | - |  |
| HLA-DR+CD38+ | - | 0.0047 | 0.0107 | - | - | - |  |
| PD-1+ | - | - | - | - | - | - |  |
| CD39+ | 0.0187 | 0.0024 | 0.0173 | - | - | - |  |
| CD73+ | - | - | - | - | - | - |  |
| **TEMRA** |  |  |  |  |  |  |  |
| CCR5+ | - | - | - | - | - | - |  |
| HLA-DR+CD38+ | - | 0.0039 | 0.0007 | - | - | - |  |
| PD-1+ | - | - | - | - | - | - |  |
| CD39+ | 0.0024 | 0.0401 | 0.0313 | - | - | - |  |
| CD73+ | - | - | - | - | - | - |  |


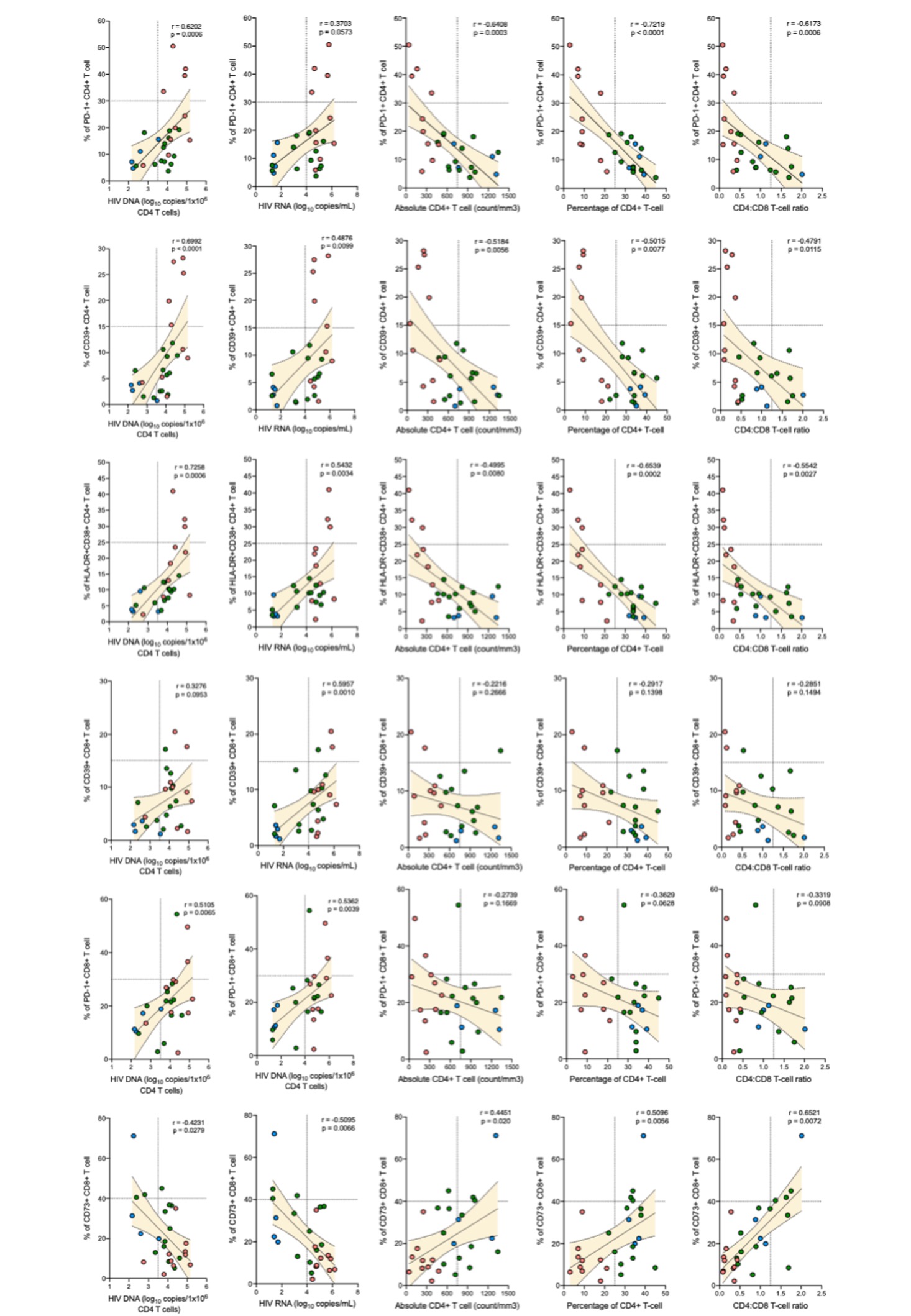


**Supplemental Figure 5.** Correlation of total HIV-DNA and HIV-RNA levels, absolute and relative CD4+ T-cell and CD4:CD8 ratio with different markers Spearman rank tests were used for correlations. Best-fit line and 95% confidence bands are shown.

**Supplemental Table 4. HLA class I alleles for 7 PECs.** In grey is highlighted the HLA-B alleles associated with viraemic control and in red those associated with rapid disease progression in adults infected with HIV.

|  | **HLA Class I** | | | | | |
| --- | --- | --- | --- | --- | --- | --- |
|  | **A1** | **A2** | **B1** | **B2** | **C1** | **C2** |
| **PEC-01** | 03:01 | 33:03 | 35:01 | 53:01 | 04:01 | 16:01 |
| **PEC-02** | 03:01 | 30:02 | 14:01 | 15:10 | 03:04 | 08:02 |
| **PEC-03** | 02:01 | 74:01 | 27:03 | 42:01 | 02:02 | 07:01 |
| **PEC-04** | 03:01 | 68:02 | 14:02 | 58:02 | 06:02 | 08:02 |
| **PEC-05** | 03:01 | 30:02 | 18:01 | 57:03 | 07:01 | 07:04 |
| **PEC-06** | 23:01 | 68:02 | 08:01 | 14:01 | 03:04 | 08:02 |
| **PEC-07** | 23:01 | 74:01 | 15:16 | 53:01 | 04:01 | 14:02 |


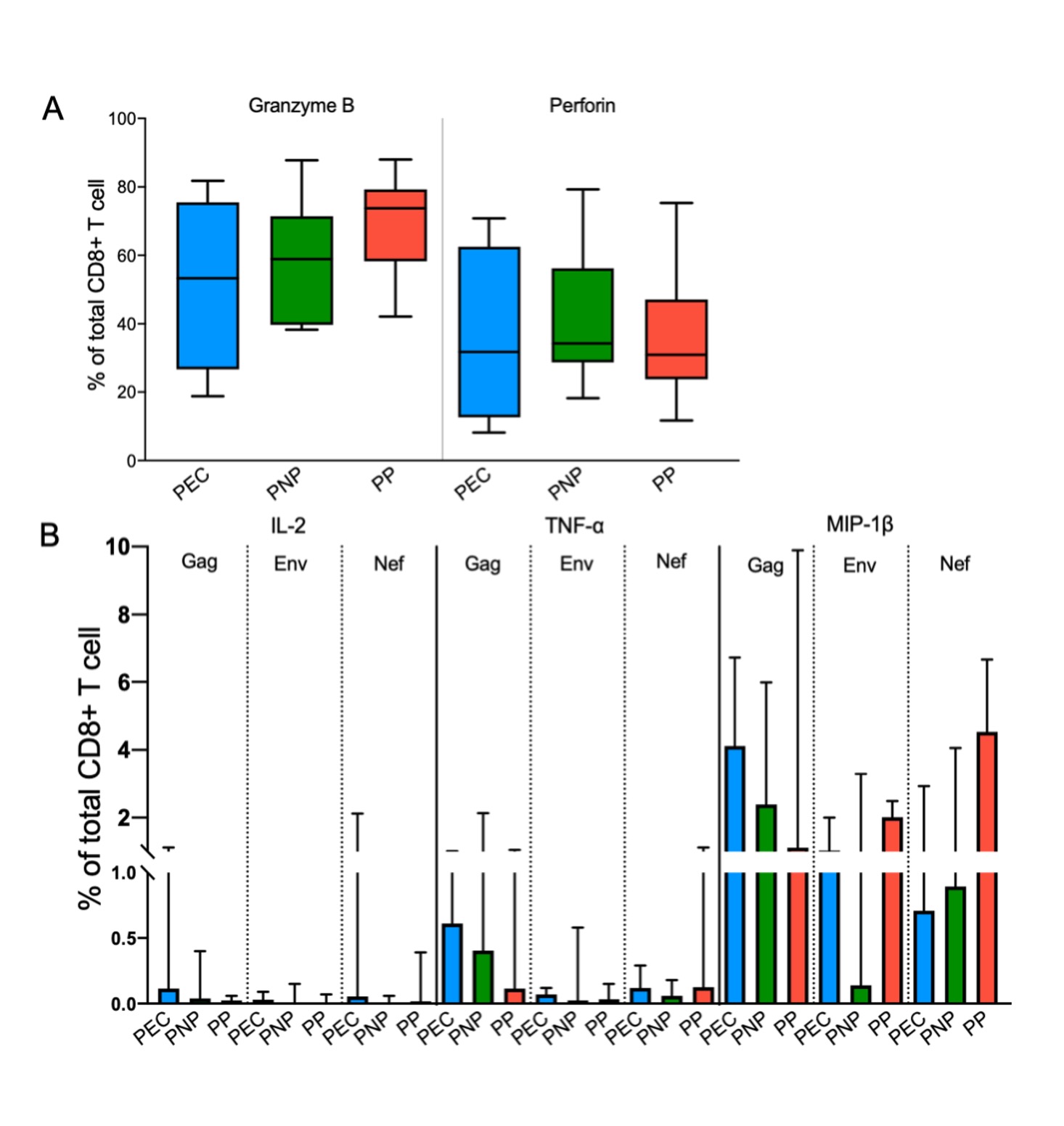


**Supplemental Figure 6.** A. Expression of Granzyme B and Perforin in total CD8+ T-cells in each group. B. Expression of IL-2, TNF-α and MIP-1β in total CD8+ T-cell in each group upon stimulation with Gag, Env and Nef HIV clade C pools. For cytokines, the values are after background subtraction. The plots are expressed in median and interquartile. Comparisons were done with Kruskal-Wallis’ test.
